# Investigation and countermeasure of the dietary nutrition status of college students from the perspective of healthy China

**DOI:** 10.1038/s41598-026-36178-x

**Published:** 2026-01-16

**Authors:** Yu Yuan, Xiaoyu Liu, Shuting Yang, Yaya Liu

**Affiliations:** Shanxi College of Applied Science and Technology, Taiyuan, 030062 Shanxi China

**Keywords:** Healthy China, College students, Dietary nutrition, KAP, BMI, Nutrition, Occupational health, Paediatric research

## Abstract

This study examined dietary nutrition knowledge, attitudes, and practices (KAP) among college students and proposed improvement strategies within the framework of Healthy China. A questionnaire survey was conducted among 815 students, and correlation and inferential statistical analyses were performed. Results revealed significant positive correlations among nutrition knowledge, attitudes, and practices (*p* < 0.01). The total KAP score was strongly correlated with knowledge (*r* = 0.825), attitudes (*r* = 0.492), and practices (*r* = 0.742). Females scored higher than males in nutrition knowledge and attitudes, while no significant sex difference was found in dietary practices. Only 54.97% of students had a normal BMI. In descriptive analyses, students with normal weight tended to have higher mean scores in attitudes, practices, and total KAP, although these differences were not statistically significant. The average knowledge (7.74 ± 2.81) and practices (10.43 ± 2.37) scores were relatively low, indicating knowledge deficits and poor dietary practices, although attitudes were positive (3.61 ± 0.86). Universities should strengthen nutrition education through digital platforms (e.g., social media, apps, short videos) to enhance students’ nutritional literacy and promote healthier dietary practices.

## Introduction

Nutrition refers to the process by which humans obtain, digest, absorb, and utilize food to meet physiological needs^1^. Nutrition serves as the material basis for life activities and is a crucial factor for ensuring the normal growth and development of the human body. Among the various factors influencing growth and development, nutrition plays a vital role^2^. College students are in a critical stage of growth and life development, facing significant pressures related to study, employment, social interactions, and physical well-being^3^. The demand for various nutrients is high; however, the current nutritional status of college students is concerning, with both malnutrition and overnutrition being prevalent^4^. The Dietary Nutrition Knowledge, Attitudes, and Practices (KAP) questionnaire is an effective measurement tool for assessing the impact of nutritional education and interventions. It has a wide range of applications, including nutritional surveys and interventions targeting different populations^5^. Studies using the KAP questionnaire to investigate the knowledge and attitudes of athletes and college students have shown that while athletes exhibit relatively positive nutritional attitudes, they often lack adequate nutritional knowledge^6^.

In KAP studies, body mass index (BMI) is a critical indicator widely used to assess individual nutritional status and physical development levels^7^. Generally, a BMI between 18.5 and 24 kg/m^2^ is considered normal; a BMI below 18.5 kg/m^2^ indicates underweight; a BMI of 24–27.9 kg/m^2^ indicates overweight; and a BMI ≥ 28 kg/m^2^ is classified as obesity^8^. Previous studies have indicated that college students find maintaining a health-promoting lifestyle challenging because of various factors, such as excessive stress, busy schedules, and lifestyle changes^9^. Poor dietary patterns have contributed to adverse health outcomes among Chinese college students, including obesity and impaired immunity^10^. Barriers such as lack of knowledge or information, lack of interest in changing diets, financial constraints, time limitations, or taste preferences may hinder individuals from adopting healthier eating habits^11^. While the relevant literature has extensively discussed the flaws and causes of dietary structures, comprehensive recommendations for improving research outcomes are lacking.

This study investigates dietary nutrition knowledge, attitudes, and practices among college students, with a focus on differences by sex and BMI. It aims to provide a comprehensive understanding of students’ nutritional cognition and behaviors—including dietary habits, knowledge sources, and daily health practices—and to identify gaps across sex and BMI categories to inform evidence-based strategies for promoting balanced nutrition and health literacy.

## Methods

### Survey subjects

The study targeted 815 college students, including 301 males (36.9%) and 514 females (63.1%). A KAP questionnaire on dietary nutrition was administered to the students. All participants were self-reported as free from major chronic diseases during the survey period. Independent sample *t*-tests revealed significant differences between male and female students in terms of age, height, and weight (*p* < 0.01), with specific data detailed in Table 1. All methods were carried out in accordance with relevant guidelines and regulations. The experimental protocols were reviewed and approved by the Ethics Committee of Shanxi Applied Science and Technology College. Informed consent was obtained from all subjects involved in the study, and from their legal guardian(s) if participants were under 18 years of age.

**Table 1:** Overview of basic characteristics of the study subjects.

**Variable**	**Males (n = 301)**	**Females (n = 514)**	**p values**
Age (years)	21.25 ± 1.31	20.58 ± 1.10	< 0.01
Height (cm)	178.34 ± 5.80	164.56 ± 6.97	< 0.01
Weight (kg)	72.86 ± 15.10	53.97 ± 9.44	< 0.01

Note: Data are presented as mean ± standard deviation. p values were calculated using two-tailed independent-samples *t*-tests to compare differences between male and female students.

### Questionnaire survey

On the basis of the research objectives and referencing domestic and international dietary nutrition questionnaires, a KAP questionnaire for college students was designed^12^. A cross-sectional study was conducted using a convenience sample of undergraduate students recruited from Shanxi Applied Science and Technology College. Faculty members assisted in participant recruitment. The questionnaire was administered electronically via the WeChat social media platform, with responses collected in real-time using the Wenjuanxing survey tool. The data collection process comprised three sequential phases: questionnaire development and piloting (June 2024), survey distribution and data acquisition (July 2024), and data analysis (August 2024).

The questionnaire consisted of two modules. The first module collected basic demographic information, including sex, age, height, and weight. The second module assessed dietary nutrition KAP, comprising three sections: knowledge (7 items on sources of essential nutrients and nutrient comprehension), attitudes (4 items on willingness to change unhealthy eating habits and the importance of nutrition education), and practices (19 items on food intake frequency, daily dietary combinations, and related health behaviors), totaling 30 items.

The validity and reliability of the questionnaire were tested. Using expert validity evaluation, six experts reviewed the questionnaire, with four rating it as valid and two rating it as very valid, indicating strong relevance, a reasonable design, and understandable options and suggesting high validity. A Cronbach’s alpha test yielded an initial coefficient of 0.726. After removing low-correlation items, 200 participants were randomly selected from the original sample of 815 for a test–retest assessment 20 days later, with stratified random sampling by age to preserve proportional representation. Only students who completed both surveys were included. The resulting Cronbach’s alpha of 0.828 indicated high reliability, and the retest sample retained demographic characteristics comparable to the original cohort.

### Statistical analysis

A comprehensive statistical analysis was conducted on the questionnaire data, including descriptive statistics, correlation analysis, and significance testing. Descriptive statistics were calculated for all study variables. Categorical data (sex) are presented as frequencies and percentages. Continuous variables (age, height, weight, and BMI) are presented as mean ± standard deviation. Independent samples Student’s *t*-tests were used to assess sex differences in age, height, and weight. Pearson correlation coefficients were calculated to examine relationships among sex, height, weight, BMI, dietary nutrition knowledge, attitudes, practices, and total KAP scores.

For comparisons by sex, Levene’s test was first performed to assess homogeneity of variances. When the assumption of equal variances was met (*p* > 0.05), the standard independent-samples *t*-test was applied; otherwise (*p* < 0.05), Welch’s *t*-test was used.

For comparisons across BMI groups, participants were categorized into three groups: underweight (BMI < 18.5 kg/m^2^), normal weight (18.5 ≤ BMI < 24 kg/m^2^), and overweight/obese (BMI ≥ 24 kg/m^2^). Due to the relatively small number of obese participants, overweight and obese students were combined into a single group (BMI ≥ 24 kg/m^2^) for statistical analysis. Levene’s test was conducted for each variable to assess variance homogeneity. Variables with homogeneous variances were analyzed using one-way ANOVA (*F*-test), whereas variables with unequal variances were analyzed using Welch’s ANOVA. Post hoc pairwise comparisons among BMI groups were performed using Bonferroni correction to adjust for multiple comparisons and control the family-wise error rate.

All statistical analyses were performed using SPSS version 27.0 (IBM Corp., Armonk, NY, USA). Variable assignments are presented in Table 2.

**Table 2:** Assignment of analytical factors.

**Factor**	**Coding (Numeric assignment)**		
**Sex**	Male = 1	Female = 2	
Height (cm)	150–168 = 1	169–186 = 2	187–200 = 3
Weight (kg)	35–70 = 1	71–105 = 2	106–140 = 3
BMI (kg/m^2^)	<18.5 = 1	18.5 ≤ BMI < 24 = 2	≥24 = 3

Note: Numeric codes were used for subsequent statistical analyses. Height and weight were categorized into tertiles based on the sex-specific distribution within the study sample to ensure balanced group sizes for comparative analyses. BMI was categorized according to the Chinese adult standard: underweight (<18.5 kg/m^2^), normal weight (18.5–23.9 kg/m^2^), and overweight/obesity (≥24.0 kg/m^2^).

### Evaluation criteria

The scoring for the dietary nutrition knowledge section was based on the *Dietary Guidelines for Chinese Residents* (2022), with a total score of 14 points^13^. For single-choice questions, a correct answer earns 1 point, whereas for multiple-choice questions, each correct answer earns 1 point. The dietary nutrition attitudes section has a total score of 4 points, with a score of 1 for positive or very positive attitudes and 0 otherwise. The dietary nutrition practices section has a total score of 19 points, with a score of 1 for choosing appropriate dietary nutrition practices and 0 otherwise. The combined maximum score for all three sections is 37 points. A score equal to or greater than 60% of the total was required to pass. Performance was classified into five levels (fail, pass, moderate, good, and excellent), with specific evaluation standards detailed in Table 3.

**Table 3:** Dietary nutrition KAP evaluation standards.

**Item**	**Fail**	**Pass**	**Moderate**	**Good**	**Excellent**
Dietary nutrition knowledge	[0, 8.4)	[8.4, 9.8)	[9.8, 11.2)	[11.2, 12.6)	[12.6, 14.0]
Dietary nutrition attitudes	[0, 2.4)	[2.4, 2.8)	[2.8, 3.2)	[3.2, 3.6)	[3.6, 4.0]
Dietary nutrition practices	[0, 11.4)	[11.4, 13.3)	[13.3, 15.2)	[15.2, 17.1)	[17.1, 19.0]

Note: KAP = Knowledge, Attitudes, and Practices. Values represent the scoring ranges for each level, with 60% of the total score used as the minimum threshold.

## Results

### BMI index of survey participants

**Fig. 1:**
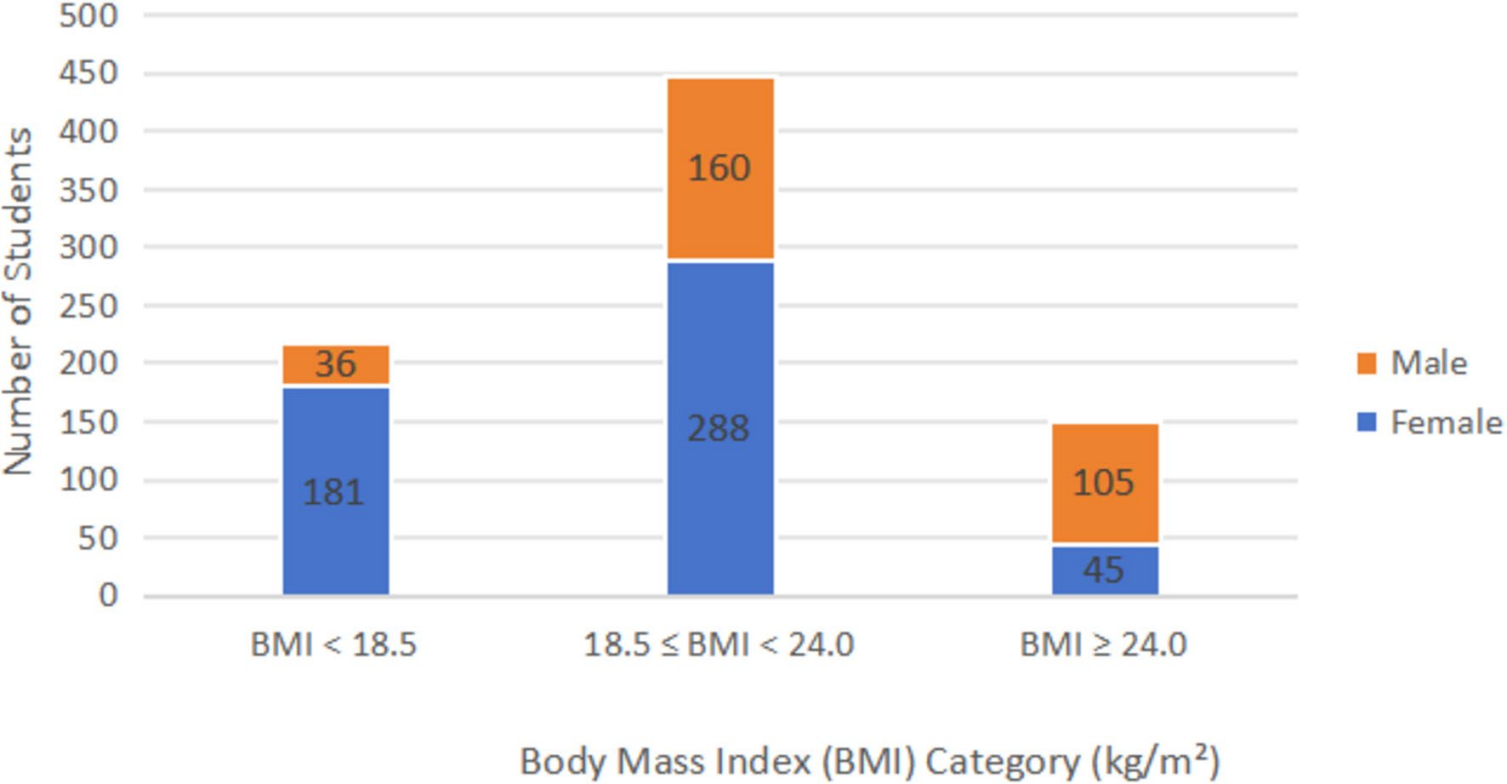
Distribution of students across underweight, normal weight, and overweight/obese body mass index (BMI) categories.

According to the *Global Nutrition Report*, the prevalence of overweight and obesity among Chinese adults aged 18 years and above has shown a steady upward trend in recent years^14^. As shown in Figure 1, analysis of BMI among 815 college students showed that 26.63% were underweight, 54.97% had a normal BMI, and 18.40% were overweight or obese. The mean BMI within each category was 17.20 kg/m^2^ (SD = 1.12) for underweight, 20.98 kg/m^2^ (SD = 3.66) for normal weight, and 26.78 kg/m^2^ (SD = 3.06) for overweight/obese students. Sex-specific analysis revealed a statistically significant difference in the distribution across BMI categories between males and females (*χ*^2^ = 17.28, *p* < 0.01). Specifically, underweight status was significantly more prevalent among females, whereas overweight or obesity was notably more common among males.

### Correlation analysis of dietary nutrition KAP and other variables

As shown in Table 4, correlation analysis revealed significant positive associations among dietary nutrition knowledge, attitudes, and practices, supporting the interrelated structure of the KAP model among college students. Nutrition knowledge was positively correlated with dietary attitudes (*r* = 0.294, *p* < 0.01) and dietary practices (*r* = 0.275, *p* < 0.01), while attitudes were also correlated with practices (*r* = 0.225, *p* < 0.01). Because multiple correlations were tested, Bonferroni correction was applied for the three pairwise correlations among knowledge, attitudes, and practices (adjusted α = 0.0167); all associations remained statistically significant after adjustment, confirming the robustness of these relationships.

**Table 4:** Correlation data overview of dietary nutritional KAP.

**Indicator**	**Sex**	**Knowledge**	**Attitudes**	**Practices**	**Height**	**Weight**	**BMI**	**KAP Total score**
Sex	1							
Knowledge	.082^*^	1						
Attitudes	.077^*^	.294^**^	1					
Practices	-.009	.275^**^	.225^**^	1				
Height	-.722^**^	-.136^**^	-.087^*^	-.029	1			
Weight	-.523^**^	-.016	-.048	-.012	.449^**^	1		
BMI	-.368^**^	-.022	-.008	-.012	.251^**^	.587^**^	1	
Total score	.061	.825^**^	.492^**^	.742^**^	-.117^**^	-.025	-.035	1

Note: *r* indicates the Pearson correlation coefficient. *p* values are two-tailed. *p* < 0.05 (*) and p < 0.01 (**) denote statistical significance. Sex was coded as 1 = Male, 2 = Female. Correlations involving sex were interpreted as point-biserial correlations. KAP Total Score = Knowledge + Attitudes + Practices.

Regarding physiological characteristics, males were generally taller and heavier and had higher BMI values than females. Height showed a weak negative correlation with nutrition knowledge (*r* = -0.136, *p* < 0.01) and attitudes (*r* = -0.087, *p* < 0.05). Overall, knowledge, attitudes, and practices were strongly correlated with the total KAP score (*r* = 0.825, 0.492, and 0.742, respectively; all *p* < 0.01).

### Significance analysis of dietary nutritional KAP and other variables

**Fig. 2:**
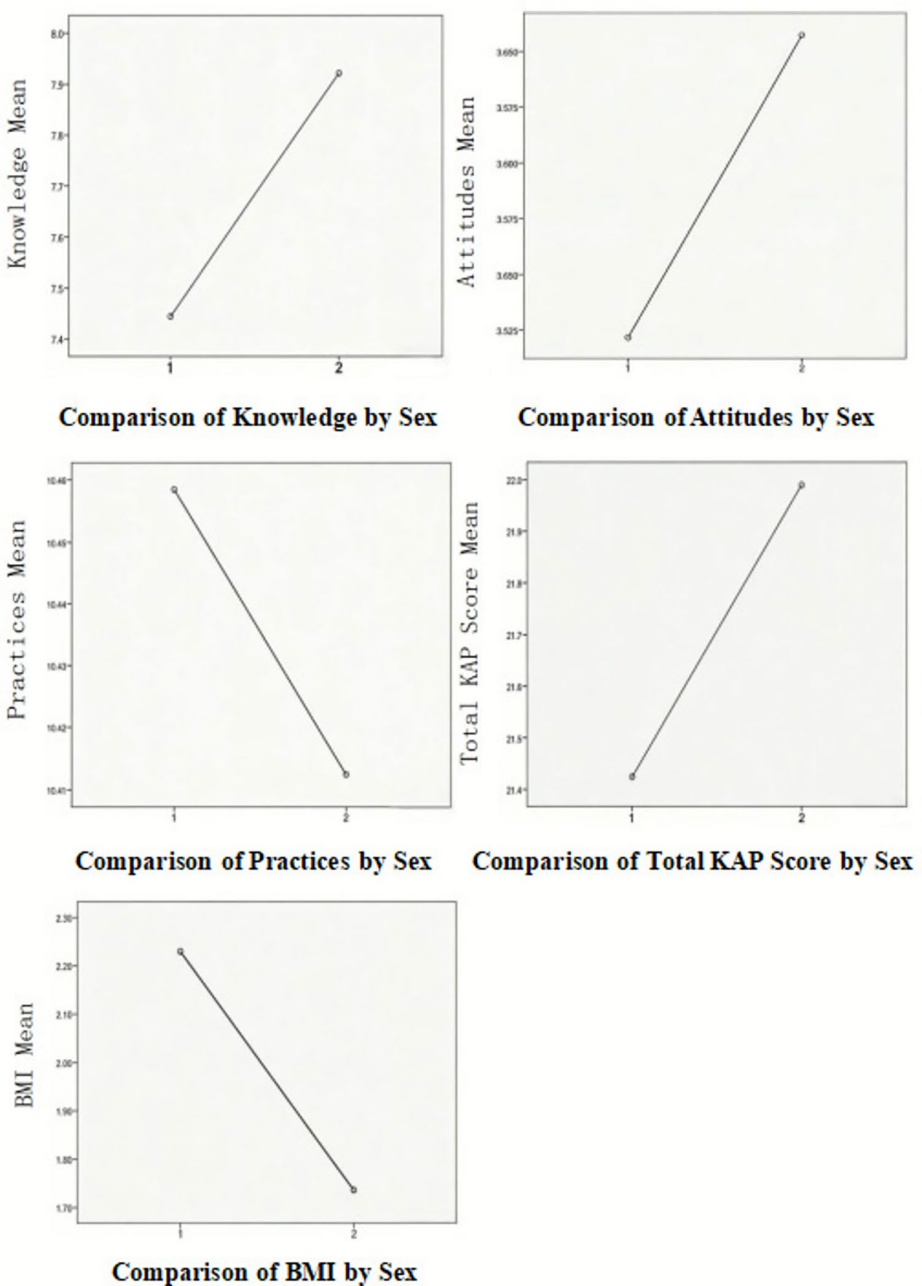
Comparisons of mean dietary nutrition knowledge, attitudes, practices, total KAP score, and BMI by sex. KAP, knowledge, attitudes, and practices. Sex codes: 1 = Males, 2 = Females.

Table 5 and Figure 2 illustrate the comparative trends across sexes. Levene’s test was conducted to evaluate the homogeneity of variances. Independent-samples Student’s *t*-test or Welch’s *t*-test were performed as appropriate. Homogeneous variance was identified for dietary practices (*p* = 0.094), allowing the use of an independent-samples *t*-test. Variances were unequal for dietary knowledge, attitudes, total KAP score, and BMI (*p* < 0.05), requiring Welch’s *t*-test.

**Table 5:** Significance of Sex, dietary nutritional KAP, total KAP score, and BMI

**Indicator**	***t***	**df**	***p*-value**	**Mean difference**
Knowledge^*^	-2.277	577.745	0.023	-0.475
Attitudes^*^	-2.089	538.186	0.037	-0.136
Practices	0.267	813	0.790	0.046
Total score^*^	-1.680	569.633	0.094	-0.565
BMI^*^	10.648	484.337	< 0.001	2.851

Note. Mean difference was calculated as Male mean minus Female mean; a positive value indicates a higher mean for males, and a negative value indicates a higher mean for females. Variables marked with an asterisk (*) were analyzed using Welch’s *t*-test due to heterogeneous variances (Levene’s test *p* < .05); all other variables were tested using the standard independent-samples (Student’s) *t*-test.

The results indicated no significant differences between male and female students in dietary practices (*t*(813) = 0.267, *p* = 0.790) or total KAP score (Welch’s *t*(569.63) = -1.680, *p* = 0.094). Significant differences were observed in dietary knowledge (Welch’s *t*(577.75) = -2.277, *p* = 0.023), dietary attitudes (Welch’s *t*(538.19) = -2.089, *p* = 0.037), and BMI (Welch’s *t*(484.34) = 10.648, *p* < 0.001).On average, female students scored higher than males in dietary knowledge and attitudes, whereas males had higher BMI values.

### Significance analysis of variables by different BMI groups

**Fig. 3:**
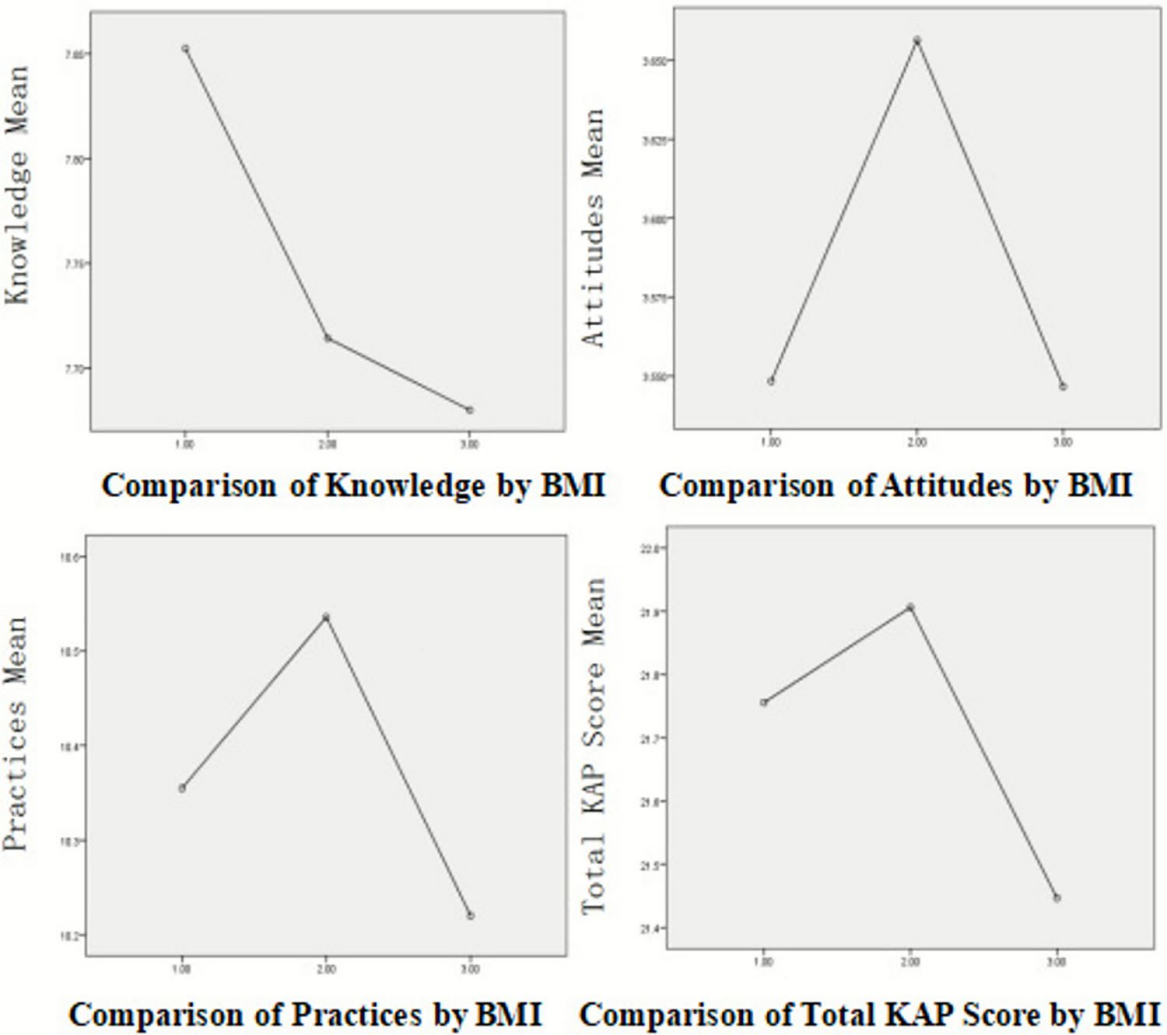
Comparisons of mean dietary nutrition KAP scores and total score across body mass index (BMI) categories. KAP, knowledge, attitudes, and practices. Categories: 1 = underweight, 2 = normal weight, 3 = overweight/obese.

The results are summarized in Table 6 and Figure 3, following the comparison of dietary nutrition knowledge, attitudes, practices, and total KAP scores across three body mass index (BMI) categories: underweight (BMI < 18.5 kg/m^2^, n = 217), normal weight (18.5 ≤ BMI < 24 kg/m^2^, n = 450), and overweight/obese (BMI ≥ 24 kg/m^2^, n = 148).

**Table 6:** Results of ANOVA comparing dietary nutrition KAP scores across BMI groups.

**Indicator**	**Test Used**	***F / H***	***p*-value**
Knowledge	One-way ANOVA	*F = 0.098*	*0.226*
Attitudes	Welch’s ANOVA	*F =* 1.592	0.205
Attitudes (Robustness check)	Kruskal-Wallis *H* Test	*H* = 3.486	0.175
Practices	One-way ANOVA	*F =* 1.432	0.141
Total Score	One-way ANOVA	*F =* 1.019	0.591

Note: The choice between standard one-way ANOVA and Welch’s ANOVA was based on Levene’s test for equality of variances. A non-parametric Kruskal-Wallis *H* test was conducted for dietary attitudes as a robustness check due to significant heteroscedasticity.

Levene’s test indicated a significant violation of the homogeneity of variance assumption for dietary attitudes (*p* = .006). Therefore, Welch’s ANOVA was applied for this variable, while standard one-way ANOVA was used for knowledge, practices, and total score, which met the variance homogeneity assumption. The analysis revealed no statistically significant differences across BMI groups in dietary nutrition knowledge (*F* = 0.098, *p* = .226), attitudes (Welch’s *F* = 1.592, *p* = .205), practices (F = 1.432, *p* = .141), or total KAP scores (*F* = 1.019, *p* = .591).

As a robustness check for dietary attitudes given its heteroscedasticity, a non-parametric Kruskal-Wallis H test was conducted. The results corroborated the ANOVA findings, indicating no significant difference in attitude scores across BMI groups (*H* = 3.486, *p* = .175). At the descriptive level, students with normal weight tended to have higher mean scores in attitudes, practices, and total KAP, while underweight students showed slightly higher knowledge scores, although these differences were not statistically significant. These descriptive tendencies should be interpreted with caution, as no statistically significant differences were observed across BMI groups.

### Dietary nutrition knowledge survey data analysis

**Fig. 4:**
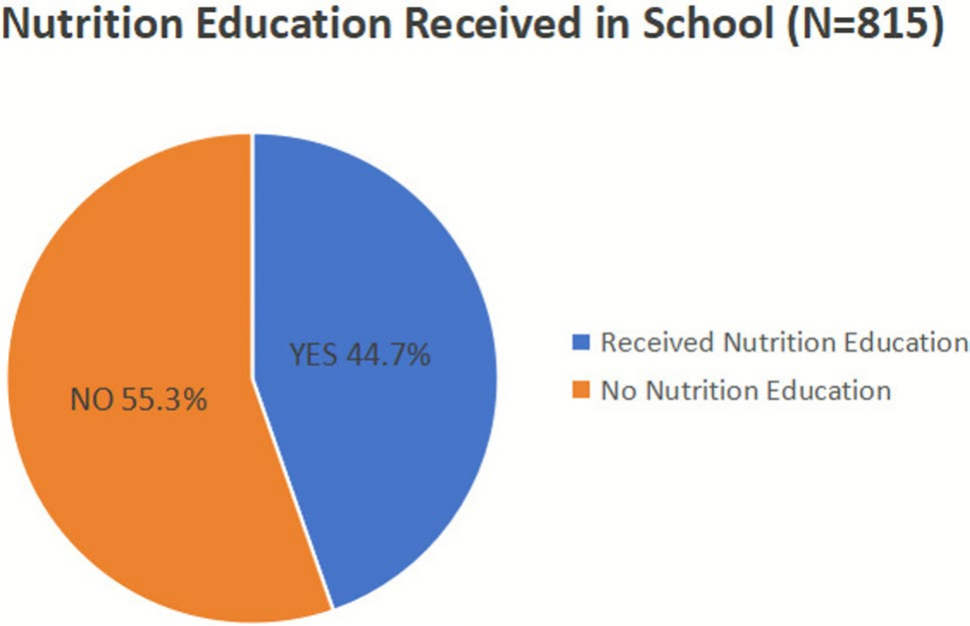
Proportion of college students reporting receipt of systematic nutrition education during their school years. Among 815 respondents, 364 students (44.7%) reported having received systematic nutrition education, while 451 students (55.3%) reported not having received such education.

As shown in Figure 4, statistical analysis revealed significant positive correlations between dietary nutrition knowledge and both dietary attitudes and practices, indicating that higher knowledge scores were associated with more positive attitudes and healthier practices. The mean dietary nutrition knowledge score among college students was 7.74 ± 2.81, which was below the recommended threshold. Female students scored significantly higher than male students in dietary knowledge (7.92 ± 2.69 vs. 7.45 ± 2.98; Welch’s *t*(577.75) = -2.277, *p* = 0.023). The lowest score was 1 (with 3 students), and only 13 students achieved the highest score of 14, indicating variability in nutrition knowledge among students.

**Fig. 5:**
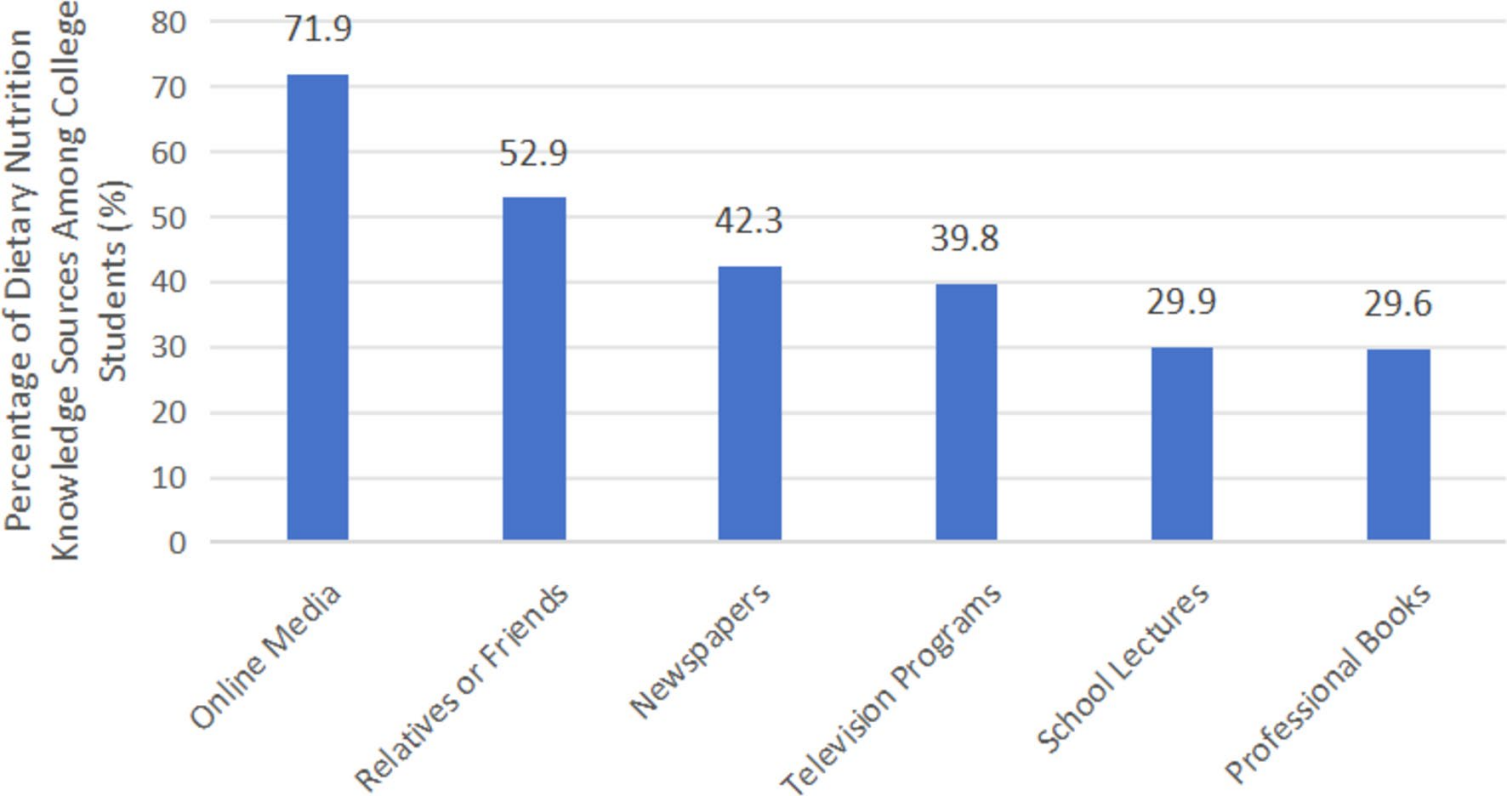
Percentage of college students reporting various pathways for acquiring nutrition knowledge. Values represent the proportion of students who identified each channel as a source of nutritional information.

As shown in Figure 5, analysis of the survey data revealed that online media was the most frequently reported source of nutrition knowledge among university students, followed by relatives or friends. Traditional media sources such as newspapers and television programs were less commonly used, while school lectures and professional books were the least utilized, indicating limited reliance on formal educational channels. Regarding the perceived reliability of nutrition information, a substantial proportion of students were uncertain or skeptical, suggesting doubts about the credibility of media-based nutrition information.

**Fig. 6:**
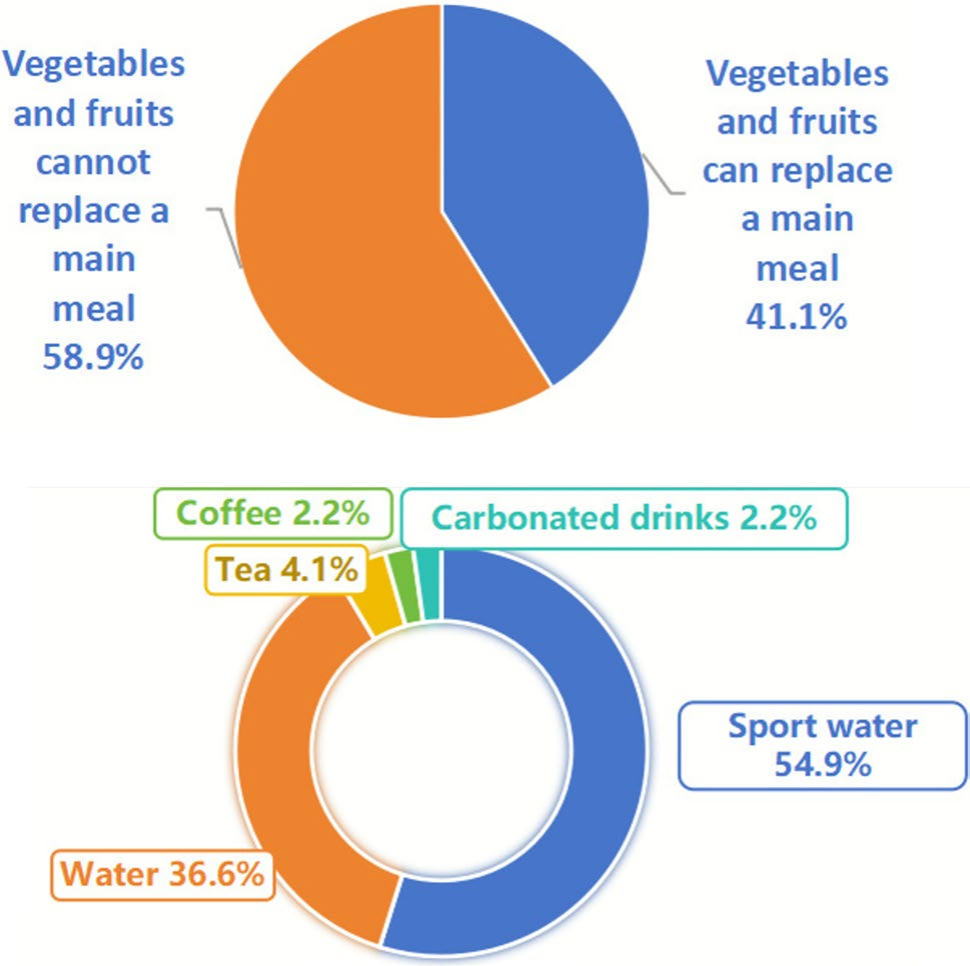
Distribution of student responses to two dietary knowledge questions. Responses are shown for (**A**) whether vegetables and fruits can replace regular meals, and (**B**) appropriate choices for replenishment after high-intensity exercise.

As shown in Figure 6, analysis of survey responses showed that 58.9% of students believed vegetables and fruits could not replace regular meals, while 41.1% held the opposite view. Regarding post-exercise hydration, most students selected sports drinks or water, whereas only a small proportion reported choosing tea, coffee, or carbonated beverages. Semi-structured interviews indicated that some female students occasionally replaced regular meals with fruits or vegetables to lose weight, and many students reported habitual coffee consumption.

### Analysis of dietary nutrition attitudes survey data

Nutritional attitudes directly influence the desire for nutritional knowledge, and they also impact the practical process of dietary habits^15^. As shown in Table 7, statistical analysis revealed significant positive correlations among dietary nutrition attitudes, knowledge, and practices, indicating that attitudes reliably reflected students’ nutritional understanding and practical orientation. The mean dietary nutrition attitude score among college students was 3.61 ± 0.86, falling within the excellent range. Female students demonstrated significantly more positive dietary attitudes than males (3.66 ± 0.79 vs. 3.52 ± 0.95; Welch’s *t*(538.19) = -2.089, *p* = 0.037), indicating more positive attitudes toward healthy eating among females.

**Table 7:** Overview of responses to dietary nutrition attitudes survey questions.

**Content of dietary nutrition attitudes items**	Yes (%)	Not sure (%)	No (%)
Do you wish to acquire nutritional knowledge?	93.81	2.14	4.05
Is it necessary to promote nutritional knowledge in college life?	93.68	2.15	4.17
Will you make changes if unhealthy practices are pointed out?	88.40	9.02	2.58
Is it necessary to drink water if not thirsty?	80.00	9.33	10.67

Note: Values represent the proportion of respondents selecting each response. “Not sure” indicates uncertainty about the item. Percentages may not sum to 100% due to rounding.

The majority of respondents demonstrated highly positive dietary attitudes, with 645 students achieving the maximum score of 4. Most participants expressed a strong willingness to acquire nutrition-related knowledge and to modify unhealthy dietary practices, suggesting an overall favorable disposition toward adopting healthier eating practices.

### Dietary nutrition practices survey data analysis

As shown in Table 8, the mean dietary nutrition practices score among college students was 10.43 ± 2.37, below the passing threshold, indicating a prevalence of unhealthy eating habits. Male students scored slightly higher than females in dietary practices (10.46 ± 2.51 vs. 10.41 ± 2.29), but this difference was not statistically significant (*t*(813) = 0.267, *p* = .790). The lowest score was 4 and the highest 18, reflecting substantial variability in dietary practices.

**Table 8:** Overview of responses to dietary nutrition practices survey questions.

**Item**	**Daily (%)**	**More than 3 times/week (%)**	**1-3 times/week (%)**	**Never eat (%)**
Vegetables	67.12	20.61	9.94	2.33
Fruits	33.87	32.15	30.55	3.44
Dairy products	24.91	23.68	41.4	10.31
Legumes	24.29	34.72	38.77	2.21
Eggs	31.87	30.55	33.01	4.66
Seafood	4.55	10.24	78.65	6.56
Meat	41.60	35.71	20.25	2.45
Snacks	23.68	26.75	43.80	5.77
Late-night snacks	11.00	29.20	50.00	9.80
Health supplements	7.55	10.00	32.00	50.45

Note: Values represent the percentage of respondents consuming each food item at the indicated frequency. Percentages may not sum to exactly 100% due to rounding.

Comparison with the *Dietary Guidelines for Chinese Residents* (2022) revealed that most students failed to meet recommended intake levels for major food groups, including fruits, vegetables, dairy, legumes, and seafood^13^. Only one-third of participants reported daily fruit consumption, approximately one-quarter consumed dairy, and seafood intake was minimal. Vegetable and fruit intake was particularly low, suggesting unbalanced nutrient intake and potential micronutrient deficiencies. Irregular eating patterns were common, including skipping breakfast and frequent takeout orders. Daily snack consumption was reported by nearly one-quarter of students, while a minority reported regular use of health supplements. Additionally, 16% of students smoked, and over half preferred spicy foods, potentially increasing gastrointestinal risks. These findings collectively indicate suboptimal dietary practices relative to national recommendations.

**Fig. 7:**
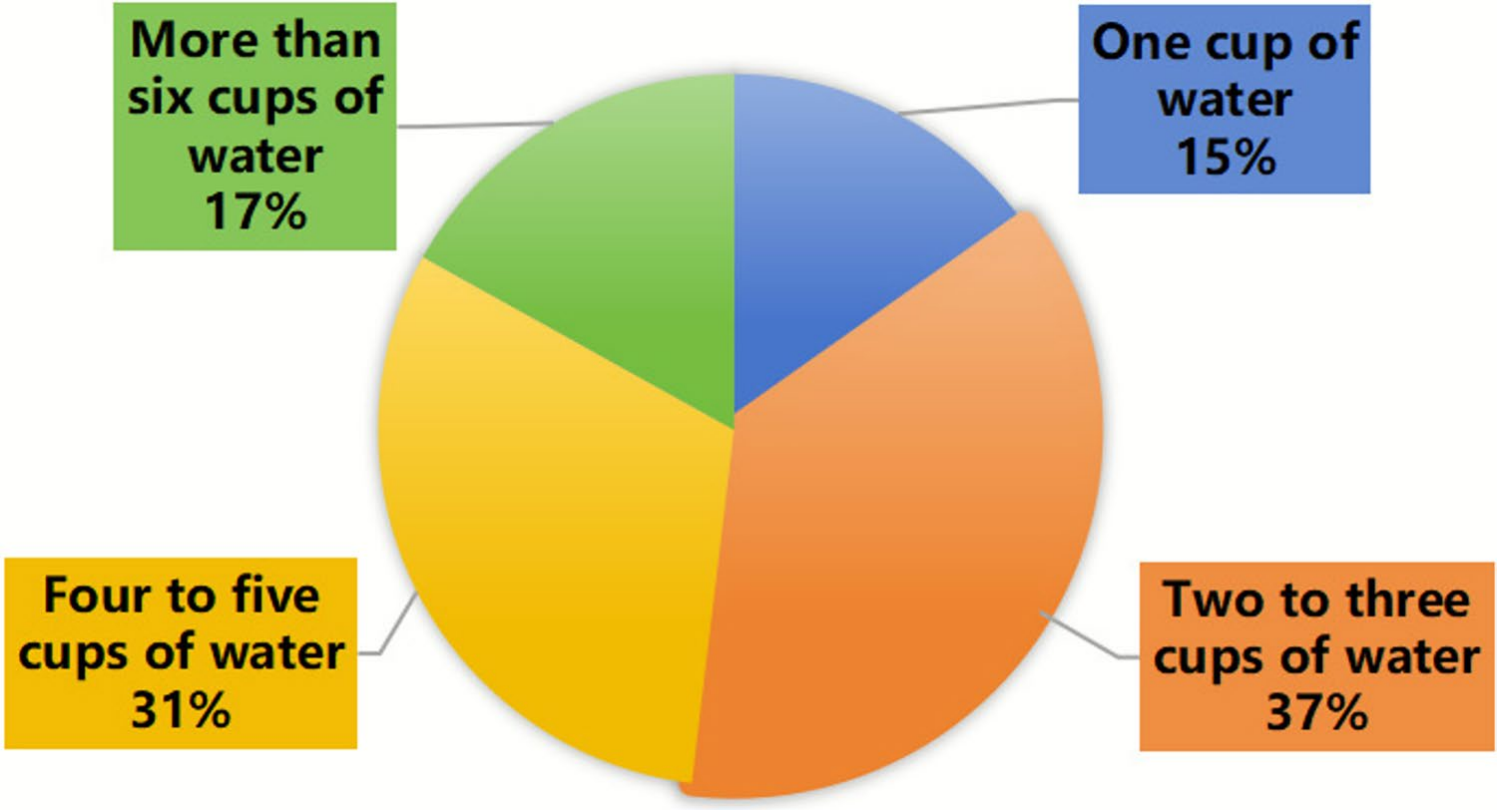
Distribution of daily water intake volumes among college students. Intake is categorized by the number of cups consumed per day. Only 17% of students met the recommended intake of more than six cups.

As shown in Figure 7, the recommended daily water intake for college students is 1500–1700 mL^13^. Survey data showed that only 17% of students met this standard. Among the participants, 15% consumed only one cup of water per day, 37% consumed 2–3 cups, and 31% consumed 4–5 cups. These results indicate that a substantial proportion of students have insufficient daily water intake.

## Discussion

The observed BMI distribution indicates that most college students maintain a normal weight, while a notable proportion are underweight or overweight/obese. Underweight was more prevalent among females, likely reflecting sociocultural pressures, restrictive diets, or lower energy expenditure, whereas overweight and obesity were more common among males, possibly due to higher caloric intake and reduced physical activity. Sex differences in weight status can be attributed to sociocultural influences, particularly the pervasive modern aesthetic ideal that equates thinness with beauty among females, along with higher levels of weight-based stigma directed toward women^16^. Together, these factors may contribute to the observed differences in weight status between female and male college students. These findings highlight the importance of sex-specific health education and nutritional interventions to promote balanced diets and adequate exercise habits.

The association between KAP and overall health status perceives a moderate positive correlation, while perceived health status shows a moderate negative correlation with knowledge and practice, indicating a link between understanding, behaviour, and health perception^17^. Female students demonstrated higher knowledge and more positive attitudes than males; however, no significant differences were observed in practices, indicating that environmental or social constraints may hinder the translation of knowledge into behavior. Weak negative correlations between height and nutrition knowledge or attitudes indicate a potential interaction between physiological and cognitive factors in shaping dietary literacy. These results emphasize the need for targeted, sex-sensitive strategies to strengthen nutrition awareness and promote effective practices.

No significant differences were observed in KAP variables across BMI groups. Normal-weight students tended to perform best in attitudes, and practices, while underweight students exhibited higher knowledge but weaker attitudes and practices—an awareness–action gap possibly linked to psychological or lifestyle factors. Although young adult men (18–25 years) are knowledgeable about nutrition, they often fail to translate this into healthy eating due to psychological (low motivation, low confidence) and lifestyle (busy schedules, cost, peer influence) barriers^18^. Although sample homogeneity may have limited between-group variability, these patterns underscore the necessity of inclusive nutrition education addressing all BMI categories.

Overall, college students demonstrated moderate nutrition knowledge, with evident gaps in energy balance and nutrient source concepts. Females scored higher than males, consistent with prior findings^19^. Most students relied on online media and social networks for dietary information, while formal education and professional literature played a minor role, raising concerns about source reliability. Survey and interview findings suggest that students’ understanding of fundamental dietary principles is limited. Some female students mistakenly perceived fruits and vegetables as adequate substitutes for complete meals, believing that restricting their intake to these foods would aid in weight control, which is inconsistent with balanced nutrition^20^. Post-exercise hydration knowledge was also suboptimal, as many students did not correctly identify appropriate fluid choices following high-intensity activity. Additionally, frequent daily coffee consumption for alertness was observed, indicating potential reliance on caffeine. These observations highlight gaps in both theoretical knowledge and practical application, underscoring the need for structured nutrition education programs to promote balanced meal planning, evidence-based hydration practices, and safe consumption behaviors^21^.

Students showed generally positive attitudes toward healthy eating, with females scoring slightly higher. Most expressed willingness to change unhealthy habits and acquire nutrition knowledge, suggesting a readiness to adopt healthier practices if provided appropriate guidance. Integrating nutrition education into health curricula and campus wellness initiatives could effectively translate favorable attitudes into sustained practical improvements.

Despite positive attitudes, students’ actual dietary practices remained suboptimal. Inadequate intake of fruits, vegetables, dairy, and seafood indicated poor adherence to the *Dietary Guidelines for Chinese Residents* (2022)^13^. Irregular meal timing, frequent snacking, and high consumption of processed or high-fat foods may exacerbate nutrient deficiencies and metabolic risks. Practices such as skipping breakfast, late-night snacking, supplement misuse, and smoking reveal persistent health literacy gaps. Although vitamins and minerals are essential, excessive supplementation without medical indication may increase metabolic burden or cause toxicity^22^. Moreover, insufficient hydration was common and may impair cognitive performance, attention, and physical endurance, emphasizing the need for education on proper fluid intake. Studies have shown that dehydration can lead to mood changes, such as reduced alertness, fatigue, and tension^23^, while acute mild hypohydration may impair vascular function and cardiovascular regulation^24^. Individuals with impaired fluid regulation, such as children, are particularly vulnerable, with dehydration potentially exacerbating early renal dysfunction^25^. Overall, universities should strengthen nutrition literacy, diversify cafeteria offerings, and ensure convenient access to safe drinking water to foster healthier dietary and hydration practices. Scientific provision of food in universities should focus on ensuring the accessibility of safe and nutritious foods and beverages, taking into account the evolving nutritional and health needs of students. Guided by principles of sports nutrition and targeting prevalent health and nutrition issues among college students, such provision emphasizes balanced diets and health promotion. By adequately supplying a variety of foods, universities can positively influence students’ dietary behaviors and overall physical health^26^.

In conclusion, Chinese college students demonstrate moderate nutrition knowledge and generally positive attitudes, yet their dietary behaviors remain suboptimal, revealing a persistent knowledge–behavior gap. Sex-specific differences in BMI, knowledge, and attitudes highlight the need for gender-sensitive interventions. Reliance on informal information sources further underscores the necessity of structured, evidence-based nutrition education. Most students primarily rely on informal sources for obtaining nutrition-related information, and many have a fairly good understanding of the basic principles of healthy eating^27^. Integrating targeted, sex-sensitive programs into campus health initiatives could effectively translate knowledge and attitudes into sustained healthy behaviors, enhance dietary literacy, support healthy weight management, and reduce nutrition-related health risks, ultimately promoting long-term physical and mental well-being.

This study relied on self-reported data, limiting causal inference and potentially introducing bias. The use of a convenience sample from a single university limits the generalizability of the findings, particularly the observed BMI distribution and the lack of significant differences across BMI groups, which might differ in a more diverse population. Dietary, hydration, and lifestyle assessments were limited, and socioeconomic and psychological confounders were not fully considered. Nutrition interventions could be extended to older students, online learners, and non-enrolled youth via digital platforms, online courses, and community workshops, but their applicability to adolescents and non-enrolled young adults requires further validation.

## Conclusions

This study provides a detailed overview of dietary nutrition among Chinese college students, revealing patterns in both nutritional knowledge and actual eating practices. Just over half of the participants (54.97%) maintained a normal BMI, with underweight status more prevalent in females and overweight more common in males, highlighting clear sex-specific disparities that are consistent with previous research in both domestic and international populations. Significant positive correlations among nutrition knowledge, attitudes, and practices indicate that greater knowledge and favorable attitudes are generally associated with healthier dietary practices. However, despite these associations, KAP scores did not differ significantly across BMI categories. Notably, underweight students exhibited higher nutrition knowledge, whereas overweight students tended to demonstrate poorer attitudes and practices, suggesting that knowledge alone may not be sufficient to promote optimal dietary practices.

Dietary practices overall remain suboptimal, characterized by irregular meal patterns, low intake of fruits, vegetables, dairy, and seafood, a high frequency of snacking, and occasional unhealthy behaviors such as skipping breakfast or late-night eating. College students encounter considerable challenges in translating nutritional knowledge into consistent healthy dietary behaviors, largely due to rapidly changing lifestyles, social influences, and limited food accessibility. Consequently, these findings underscore the need for targeted, sex-sensitive educational interventions, including campus-based nutrition programs, optimized cafeteria offerings, and guidance on balanced meal timing and healthy snacking, to enhance both knowledge and practices. Implementing such interventions could contribute to improved dietary literacy and healthier weight management, with potential benefits for reducing nutrition-related health risks and supporting long-term physical and mental well-being among college students. This study was conducted in accordance with the Declaration of Helsinki. Ethical approval was obtained from the Ethics Review Committee of Shanxi College of Applied Science and Technology. All participants provided written informed consent prior to participation.

## Data Availability

All data generated or analysed during this study are included in this published article.

## References

[1] Yuan X, Wang S, editors; Ye Y, Li D, associate editors. *Nutrition and health* Beijing: Chemical Industry Press (2017).

[2] Fu, S. Analysis of dietary nutrition problems and countermeasures among university students. *Food Ind.* **2023**, 122–124 (2023).

[3] Yanbo Z. Current situation and coping strategies of youth mental health issues. *Renmin Forum* 36–39 (2025).

[4] Ren, W. et al. Analysis and reflection on the dietary nutrition status of college students. *Modern Food* **30**, 118–121. 10.16736/j.cnki.cn41-1434/ts.2024.03.032 (2024).

[5] Sajber, D. et al. Sport nutrition and doping factors in swimming: parallel analysis among athletes and coaches. *Coll. Antropol.* **37**, 179–186 (2013). 23914506

[6] Ozdoğan, Y. & Ozcelik, A. O. Evaluation of the nutrition knowledge of sports department students of universities. *J. Int. Soc. Sports Nutr.* **8**, 11. 10.1186/1550-2783-8-11 (2011). 21892942 10.1186/1550-2783-8-11 PMC3177873

[7] Muñoz-Rodríguez, J. R., Luna, A., Gómez-Romero, F., Redondo-Calvo, F. J. & Castillo, E. The impact of biomedical education on human health and eating habits of university students in Spain. *Nutrition* **86**, 111181. 10.1016/j.nut.2021.111181 (2021). 33618137 10.1016/j.nut.2021.111181

[8] Yu, H. & He, J. The status of dietary nutrition structure of college students with obesity and its improvement measures. *Modern Food* **29**, 214–217 (2023).

[9] Kearney, J. M. & McElhone, S. Perceived barriers to trying to eat healthier: results of a pan-EU consumer attitudinal survey. *Br. J. Nutr.* **81**, 133–137 (1999). 10999038 10.1017/s0007114599000987

[10] Zinn, C., Schofield, G. & Wall, C. Evaluation of sports nutrition knowledge of New Zealand premier club rugby coaches. *Int. J. Sport Nutr Exerc Metab* **16**, 214–225 (2006). 16779927 10.1123/ijsnem.16.2.214

[11] Adams, G. M. *Exercise physiology laboratory manual* 1st edn. (McGraw-Hill, 1998).

[12] Yu, H. & He, J. Dietary nutrition structure and improvement measures for obese college students. *Modern Food* **29**, 214–217. 10.16736/j.cnki.cn41-1434/ts.2023.11.053 (2023).

[13] Chinese Nutrition Society. *Dietary guidelines for Chinese residents (2022)* (People’s Medical Publishing House, 2022).

[14] Global Nutrition Report. *Country nutrition profiles*. https://globalnutritionreport.org/resources/nutrition-profiles/ (accessed 9 August 2024).

[15] Fu Z. Effects of nutrition cognitive-practicesal intervention on nutrition improvement of adolescent swimmers [dissertation]. Shanghai: Shanghai University of Sport 10.27315/d.cnki.gstyx.2020.000304 (2020).

[16] Sattler, K. M. et al. Gender differences in the relationship between weight-based stigmatisation, motivation to exercise and physical activity in overweight individuals. *Health Psychol. Open* **5**, 1–11 (2018). 10.1177/2055102918759691 PMC5846936 29552349

[17] Bhuvaneshwari P. Nutritional knowledge, attitude, and practices among college-going students. *Journal of Current Research in Food Science* (2025).

[18] Ashton E, Hutchison M, Rollo M, et al. Motivations and barriers for young adult males to engage in healthy eating and physical activity. Conference Abstract (2014).

[19] Qiu, Y. et al. Analysis of nutrition and health knowledge levels and influencing factors among university students. *Chin J. Health Educ.* **39**, 58–63 (2023).

[20] Li X. Study on the fat loss effects of brisk walking and gene polymorphisms (adiponectin, etc.) in older adults [dissertation]. Beijing: Beijing Sport University (2009).

[21] Zhang, Z. et al. Effects of pre-exercise supplementation with different fluids on post-exercise hydration status. *Chin J. Sports Med.* **42**, 430–436. 10.16038/j.1000-6710.2023.06.003 (2023).

[22] Wan L. Health supplements can be dangerous if misused *Tianjin Daily* (005) 10.28789/n.cnki.ntjrb.2024.000985 (2024).

[23] Masento, N. A. et al. Effects of hydration status on cognitive performance and mood. *Br. J. Nutr.* **111**, 1841–1852 (2014). 24480458 10.1017/S0007114513004455

[24] Watso, J. C. & Farquhar, W. B. Hydration status and cardiovascular function. *Nutrients* **11**, 1866 (2019). 31405195 10.3390/nu11081866 PMC6723555

[25] Amaerjiang, N. et al. Dehydration status aggravates early renal impairment in children: a longitudinal study. *Nutrients* **14**, 335 (2022). 35057516 10.3390/nu14020335 PMC8778530

[26] Wang J, Lu F. Optimization of light nutritious meal provision in general higher education institutions: a multi-level fuzzy comprehensive evaluation. *High Educ Logist Res* 11–19 (2022).

[27] Geist, C. H. et al. Survey of nutrition knowledge, attitudes, and preferred informational sources among students at a southwestern university in the United States: a brief report. *Dietetics* **3**, 170 (2024).

